# A SYSTEMATIC REVIEW OF QUANTITATIVE STUDIES OF LONELINESS AND SOCIAL ISOLATION IN LONG-TERM CARE

**DOI:** 10.1093/geroni/igae098.3577

**Published:** 2024-12-31

**Authors:** LaTonya Smith, Laurie Theeke

**Affiliations:** George Washington University, Washington, District of Columbia, United States; The George Washington University, Ashburn, Virginia, United States

## Abstract

Loneliness and social isolation, though conceptually different, are both prevalent conditions for adults in long-term care facilities. These individuals, often separated from their previous lives, communities, and families, can experience a significant disconnect, leading to a pervasive sense of isolation and abandonment. Both conditions elicit a stress response, exacerbate physical health issues, lead to anxiety and depression, and significantly decrease quality of life and longevity; making it critical to understand these conditions in this population. The purpose of this paper is to present a systematic review of quantitative studies that operationalized loneliness or social isolation in samples of older adults in long-term care. The literature search used EbscoHost and included MEDLINE Complete, CINAHL complete, APA PsycArticles, and APA PsycINFO and resulted in 20 articles for inclusion. The synthesis of study findings makes it clear that loneliness and social isolation are associated with harmful health outcomes with up to 56% of residents reporting loneliness. Older adults with more functional ability and higher resilience scores were less lonely. Those with pain and those with rumination, deterministic thinking and confabulation reported increased loneliness. Intervention programs that included gardening, music, videoconferencing with families, laughter yoga, and peer mentor programs were effective for diminishing loneliness. Future studies are needed for comparative effectiveness of current interventions and for the testing of new interventions designed for this setting that are more precise to each condition. Clinical protocols for assessment and treatment of loneliness and social isolation in long-term care could enhance resident experience and potentially, longevity.

